# Exosomes in the tumor microenvironment of sarcoma: from biological functions to clinical applications

**DOI:** 10.1186/s12951-022-01609-0

**Published:** 2022-09-05

**Authors:** Huali Ye, Xin Hu, Yang Wen, Chongqi Tu, Francis Hornicek, Zhenfeng Duan, Li Min

**Affiliations:** 1 Orthopedic Research Institute, Department of Orthopedics, West China Hospital, Sichuan University, Chengdu, China; 2 West China Hospital, West China School of Medicine, Sichuan University, Chengdu, China; 3 Sarcoma Biology Laboratory, Department of Orthopaedics, Sylvester Comprehensive Cancer Center, and the University of Miami Miller School of Medicine, Miami, FL 33136 USA

**Keywords:** Exosomes, Tumor microenvironment, Sarcoma, Communication, Clinical application

## Abstract

The current diagnosis and treatment of sarcoma continue to show limited timeliness and efficacy. In order to enable the early detection and management of sarcoma, increasing attentions have been given to the tumor microenvironment (TME). TME is a dynamic network composed of multiple cells, extracellular matrix, vasculature, and exosomes. Exosomes are nano-sized extracellular vesicles derived from various cells in the TME. The major function of exosomes is to promote cancer progress and metastasis through mediating bidirectional cellular communications between sarcoma cells and TME cells. Due to the content specificity, cell tropism, and bioavailability, exosomes have been regarded as promising diagnostic and prognostic biomarkers, and therapeutic vehicles for sarcoma. This review summarizes recent studies on the roles of exosomes in TME of sarcoma, and explores the emerging clinical applications.

## Introduction

Sarcoma is a heterogeneous group of rare mesenchymal-derived cancers constituting only 1% of all malignancies [1]. However, some types, such as osteosarcoma and Ewing’s sarcoma, noticeable affect children and adolescents [2]. No specific diagnostic and prognostic indicators are available for most sarcomas, which is detrimental to the early detection and treatment evaluation [3, 4]. New diagnostic and therapeutic approaches are urgently needed to improve the overall survival of sarcoma patients.

**Fig. 1:**
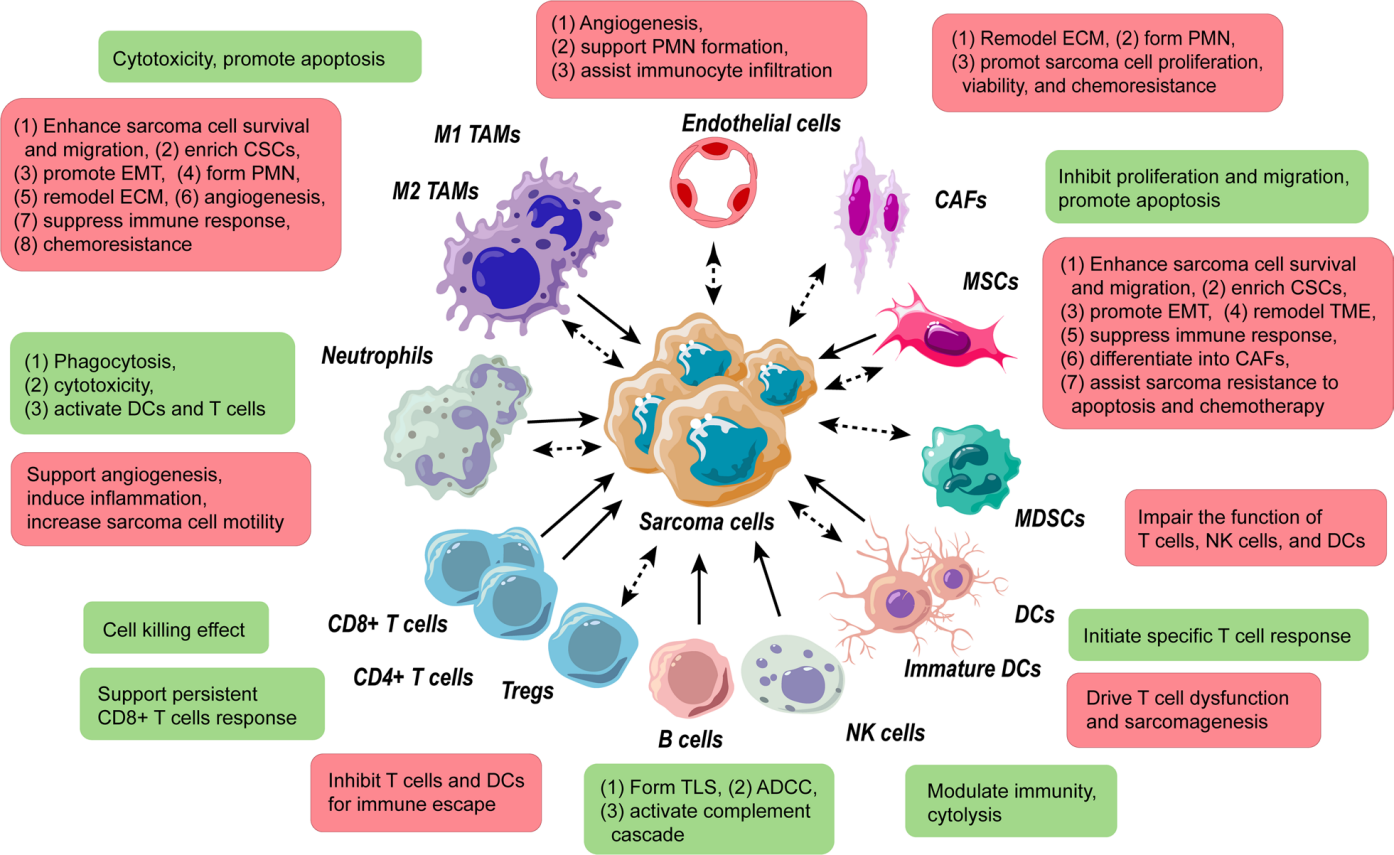
Effects of cells in TME on the progression and metastasis of sarcoma. The boxes represent the final effect on sarcoma after cellular interactions. Each red box and dotted arrow denote pro-tumor effects of microenvironment cells, and the double-head arrows indicate bidirectional actions. Green box and solid arrow represent anti-tumor effects of microenvironment cells

The tumor microenvironment (TME) is widely recognized as an essential modulator of cancer development, and a source for identifying potential biomarkers and therapies for a wide range of neoplasms, including hepatocellular carcinoma, colorectal cancer, pancreatic cancer, breast cancer, lung cancer, and sarcoma [5–10]. The TME of sarcoma encompasses sarcoma cells, immune cells, and stromal cells, whose fundamental role is to dynamically interact with sarcoma cells (Fig. 1). The interactions are mediated by contact- and noncontact-dependent mechanisms [11, 12]. The latter are connected by soluble molecules, including cytokines and subcellular structures, such as exosomes [13–17].

Exosomes, as a subtype of extracellular vesicles, are spherical particles with a diameter of 30–150 nm [18]. Exosomes are encapsulated by lipid bilayer membranes with surface proteins and enclose a cargo of biomolecules, including nucleic acids, proteins, lipids, and other bioactive substances [18–20]. The composition of exosomes varies depending on the type of parental cells. Biogenesis and secretion of exosomes are based on the endosomal system. Early endosomes are formed by the inward fusion of endocytic vesicles [21]. Early endosomes return the contents to the plasma membrane as recycling endosomes or converse into multivesicular bodies by inward invagination of endosomal membrane and cargo package into intraluminal vesicles [21]. The protein sorting of intraluminal vesicles is highly regulated by the endosomal-sorting complex required for transport-dependent or -independent pathways [22, 23]. Next, the multivesicular bodies get into the final intracellular destination, either by fusing with lysosomes to be degraded or by fusing with the plasma membrane [24]. Rab27A and Rab27B are the important mediators to induce multivesicular bodies to transfer to the cell periphery [25]. Ultimately, the soluble *N*-ethylmaleimide-sensitive factor attachment protein receptor complex drives multivesicular bodies to fuse with the plasma membrane and release intraluminal vesicles as exosomes into the extracellular microenvironment [18]. Almost all types of TME cells and malignant cells can release exosomes, which are widely distributed in biofluids [19]. After release into extracellular space, exosomes can be internalized by recipient cells through several mechanisms, including phagocytosis, macropinocytosis, plasma membrane fusion, and endocytosis [26]. Uptake mechanism and amount of exosomes by recipient cells rely on recipient cells’ surface receptors and exosomal surface proteins. Hypoxia and hypoxia-related conditions, such as low pH and oxidative stress, increase exosome production and fusion efficiency via hypoxia-induced pathways and lipid content alteration of exosome membrane [27, 28]. Exosomes have now been considered as biological vehicles transporting regulatory molecules to bridge tumor-cell interactions in TME, holding key roles in immune response, tumorigenesis, dissemination, angiogenesis, and chemotherapy resistance [29–35]. Exosomes have the properties of content specificity, cell tropism, and bioavailability in different malignancies [36, 37]. Considerable studies have demonstrated that exosomes may serve as promising non-invasive diagnostic and prognostic biomarkers, as well as possible vectors for therapeutic modalities in cancer treatment [36–39].

In this review, we summarize the most recent studies on the roles of exosomes in TME of sarcoma, and discuss the potential implications in clinic.

## The roles of exosomes in sarcoma TME

**Fig. 2:**
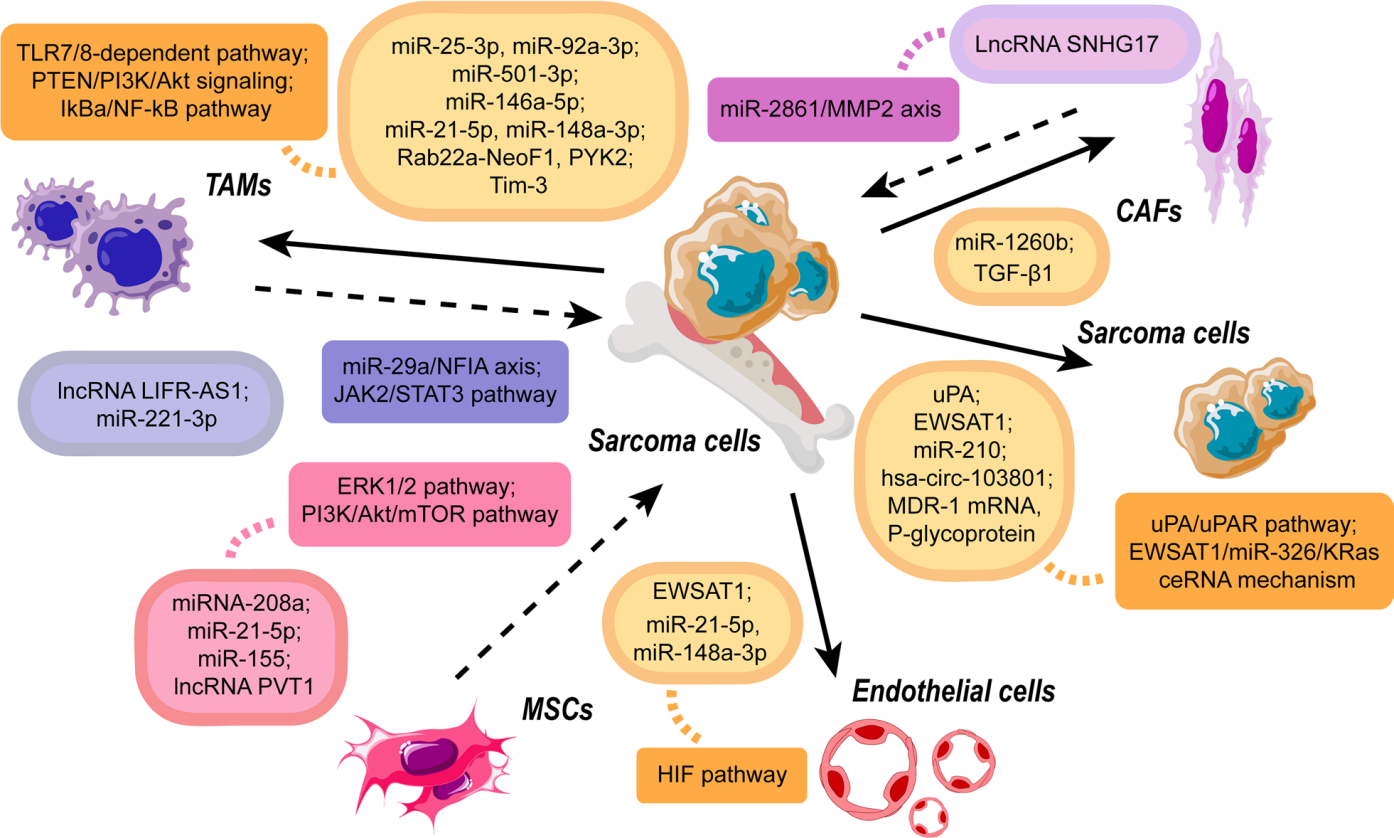
Exosomal molecules and corresponding pathways in the exosome-mediated interactions between sarcoma cells and TME cells. The dotted arrow indicates the transfer of exosomes from TME cells to sarcoma cells, while the solid arrow represents the transfer from sarcoma cells to TME cells. The oval with frame line contains the effective molecules in exosome transport. The color of each oval corresponds to the origin of the exosomes, where orange denotes sarcoma cell-derived exosomes, blue denotes TAM-derived exosomes, red denotes MSC-derived exosomes, and purple denotes CAF-derived exosomes. The box covers the corresponding pathways activated by exosomes

Exosomes are highly specialized entities for intercellular communications in TME. The main function of exosomes is to transfer bioactive substances (DNA, RNA, lipids, proteins, and other signaling molecules) from donor cells to recipient cells (Fig. 2). Exosomes provide stimulatory signals and attenuate inhibitory signals for sarcoma cells, resulting in the exchange of genetic information and reprogramming of target cells.

### Exosomes mediate the macrophage and sarcoma cell interactions for immune suppression and sarcoma progress

The development of sarcoma is linked with the immune microenvironment. Although exosomal antigens can induce tumor-specific immune reaction, accumulating studies have indicated that sarcoma creates a favorable surrounding by exosome-based dynamic signaling. Noticeably, Tumor-associated macrophages (TAMs) play key roles in shaping local immune response in TME via both promoting and suppressing immunity to sarcoma.

**Fig. 3:**
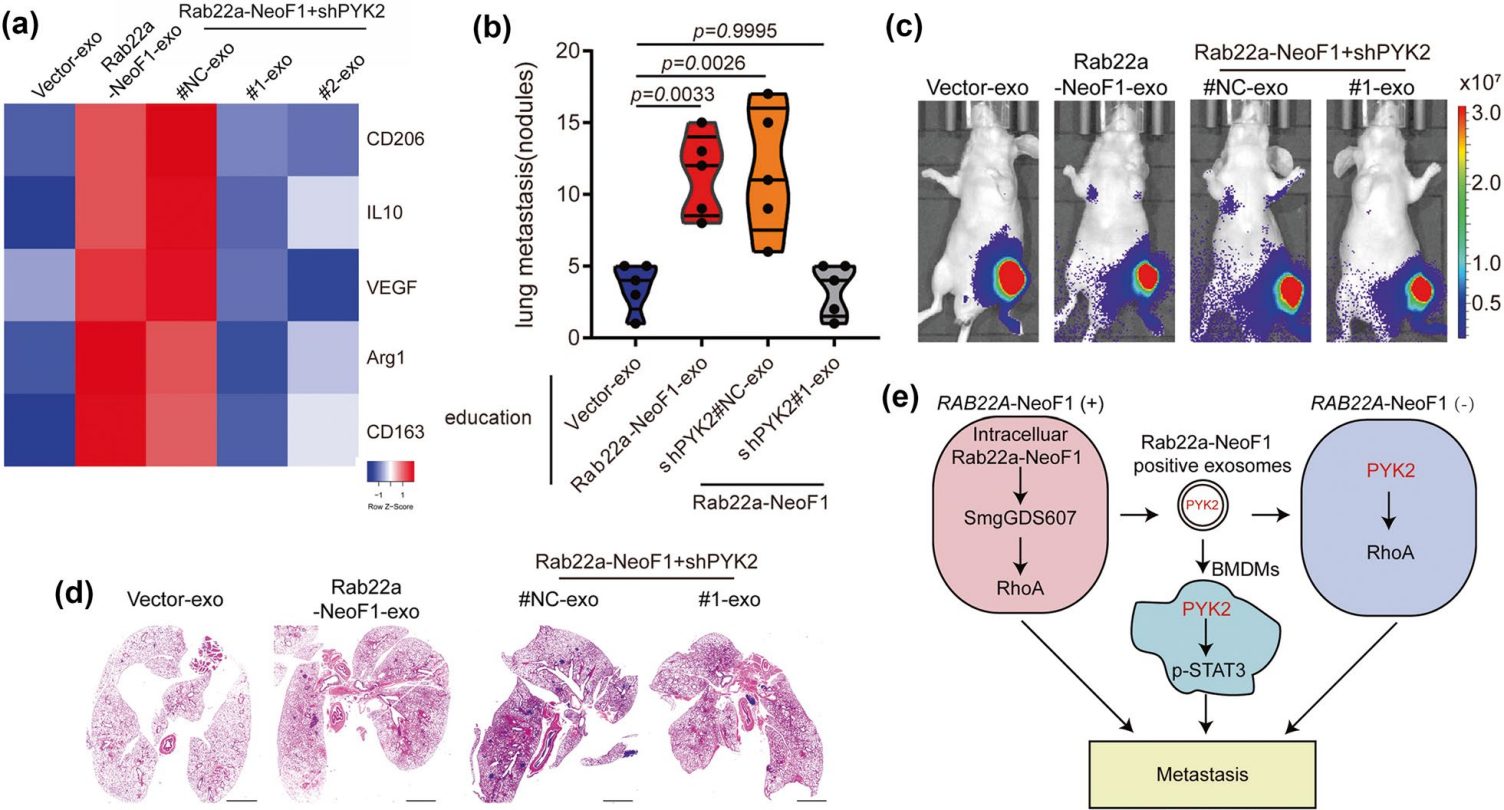
The Rab22a-NeoF1 fusion protein in exosomes facilitates M2 polarization and induces lung metastasis. **a** Macrophages were incubated with exosomes, and the heatmap showed the differential expression profiles of the M2-like macrophage markers. **b**–**d** Mice were pre-treated with exosomes for 3 weeks and subsequently injected with sarcoma cells. The lung metastases were evaluated 3 weeks later. **b** n = 5 biologically independent mice. Data are mean ± s.d with P values. **c** Representative IVIS imaging. **d** H&E-stained lung sections. **e** The hypothesis of the function and mechanism of Rab22a-NeoF1 fusion protein in osteosarcoma. Rab22a-NeoF1 fusion protein activates the cellular RhoA by binding to SmgGDS607. The exosomal Rab22a-NeoF1 promotes RhoA activation by the exosomal PYK2 and facilitates the pulmonary pre-metastatic niche formation by promoting macrophage recruitment and by increasing M2 macrophages (Reprinted with permission from Ref [14] Copyright © 2021, Li Zhong et al.)

Exosomes derived from sarcoma cells could alter the differentiation and function of macrophages, leading to local and distant spread, and immune suppression (Table 1). Sarcoma-derived exosomes promote sarcoma dissemination via targeting the mononuclear macrophage system. Exosomal microRNAs and proteins from sarcoma cells promoted the expression of cytokines and matrix metalloproteinases in macrophages [40–42]. These products endowed sarcoma cells with malignant capacity and remodeled extracellular matrix for local invasion [40–42]. Particularly, exosomes from osteosarcoma cells might affect the differentiation and maturity of osteoclasts, the specialized cells originated from monocyte/macrophage lineage [43–45]. The exosomal miR-501-3p and pro-osteoclastic contents influence osteoclastogenesis, leading to sarcoma infiltration via aggravating osteolysis [43–45]. In addition to local spread, exosomes from sarcoma are strongly associated with its distant metastasis. Exosomal miR-146a-5p suppressed osteoclastogenesis to disrupt barriers in primary lesions, which was favorable to the early progression of osteosarcoma metastasis [46]. Meanwhile, in metastatic lesions, exosomal proteins Rab22a-NeoF1 and PYK2 recruited macrophages and induced M2 polarization, establishing a functional pre-metastatic niche for pulmonary metastasis of osteosarcoma (Fig. 3) [14]. Sarcoma-derived exosomes play an essential role in these metastatic processes. Sarcoma with impaired exosome release had remarkably fewer metastatic foci and smaller areas of metastatic lesions than that with normal release ability [46]. With tumor growth, hypoxia induces sarcoma release of exosomes which can alter the immune and metabolic profile of infiltrating macrophages to better evade the immune response and promote tumor progression. Hypoxic conditions substantially enhanced the levels of immunomodulatory proteins and chemokines in exosomes produced by hypoxic tumor cells, including CSF-1, CCL2, TGF-β, FTH, and FTL [47]. The hypoxia-induced exosomes mediated monocyte/macrophage recruitment to the lesions, and the M2 phenotype was more pronounced than M1 [48]. Meanwhile, these exosomes enhanced macrophage polarization towards the M2 phenotype. The polarization may be manipulated by molecular determinants, including STAT, NF-κB, PPAR, KLF, IRF, and HIF families, and miRNAs, in which the clear role of exosomes remains to be explored. Exosomes transported let-7a miRNA and suppressed insulin-Akt–mTOR signaling pathway to enhance oxidative phosphorylation and M2 polarization of infiltrating macrophages [47]. The M2 macrophages inhibited activated CD4+ T cells and had increased the expression of mitogenic, growth, angiogenic, and pro-metastatic cytokines and enzymes [49, 50]. M2 macrophages also promoted the proliferation of tumor cells and elevated the angiogenic ability of endothelial cells by favoring oxidative phosphorylation [47].

**Table 1:** Effect of sarcoma-derived exosomes on the TME cells

Sarcoma type	TME cells	Exosomal cargos	Mechanisms	Clinical significance	References
OS	Macrophages	Tim-3	Induce conversion to M2 macrophages	Pre-metastatic lung formation↑	[40]
LPS	Macrophages	miR-25-3p, miR-92a-3p	Increase IL-6 secretion via TLR7/8-dependent pathway	Progression↑	[41]
OS	Osteoclasts; endothelial cells	miR-21-5p, miR-148a-3p	Promote bone remodeling; enhance angiogenesis	Metastasis↑	[42]
OS	Osteoblasts; osteoclasts; endothelial cells	\	Promote bone remodeling; facilitate angiogenesis via VEGF/ANGPT2/FGF2-mediated mechanism	Progression and metastasis↑	[43]
OS	Monocytes	miR-501-3p	Promote bone remodeling via PTEN/PI3K/Akt pathway	Metastasis↑	[45]
OS	Osteoclasts	miR-146a-5p	Promote bone remodeling via IkBa/NF-kB pathway	Metastasis↑	[46]
OS, FS	Monocytes	\	Transform the monocyte phenotype to inhibit CD4+ T cells	Immune suppression↑	[49]
OS	Macrophages	\	Induce conversion to M2 macrophages	Immune suppression↑	[50]
OS	Macrophages	Rab22a-NeoF1, PYK2	Activate RhoA and STAT-3	Pre-metastatic lung formation↑	[14]
EWS	CD33+ and CD14+ cells	\	Impede dendritic cell differentiation and maturation	Immune suppression↑	[55]
OS	CD11b+ Gr-1+ cells	\	Induce CD11b+ cells infiltration to lung	Pre-metastatic lung formation↑	[56]
OS	MSCs	\	Induce MSCs phenotypic conversion	Progression and metastasis↑	[58]
OS	MSCs	\	Induce LINE-1 hypomethylations for MSCs transformation	Progression and metastasis↑	[59]
MFS	Fibroblasts	miR-1260b	Decrease PCDH9 level	Progression and Infiltration↑	[67]
OS	Fibroblasts	\	Induce fibroblast phenotypic conversion	Progression↑	[68]
OS	Fibroblasts	TGF-β1	Induce myofibroblast/CAFs differentiation	Pre-metastatic lung formation↑	[70]
OS	Endothelial cells; OS cells	EWSAT1	Promote angiogenesis; regulate Akt and Erk signaling via EWSAT1/miR-326/KRas ceRNA mechanism	Progression↑	[73]

OS, osteosarcoma; miR, microRNA; LPS, liposarcoma; TLR7/8, toll-like receptor 7 and 8; VEGF, vascular endothelial growth factor; ANGPT2, angiopoietin 2; FGF2, fibroblast growth factor 2; PTEN, phosphatase and tensin homologue deleted on chromosome 10; PI3K, phosphoinositide 3 kinase; Akt, AKT8 virus oncogene cellular homolog; IkBa, inhibitor of NF-κB alpha; NF-κB, nuclear factor kappa B; FS, fibrosarcoma; RhoA, Ras homolog gene family, member A; STAT-3, signal transducer and activator of transcription 3; EWS, Ewing’s sarcoma; MSCs, mesenchymal stem cells; LINE-1, long interspersed element; MFS, myxofibrosarcoma; PCDH9, protocadherin 9; CAFs, cancer-associated fibroblasts; EWSAT1, Ewing sarcoma associated transcript 1; Erk, extracellular signal-regulated kinase; KRas, v-Ki-ras2Kirsten rat sarcoma viral oncogene homolog; ceRNA, competing endogenous RNA

TAMs, with M1-like and M2-like phenotypes, appear to be a double-edged sword in sarcoma progress via releasing exosomes (Table 2). In the early stage of sarcomagenesis, TME is predominantly infiltrated with M1-like macrophages. M1 TAMs-derived exosomes are immune-promoting and tumor-suppressive. Pro-inflammatory cytokines and cytotoxic factors were up-regulated in M1 TAMs-derived exosomes [51]. These exosomes could impede the activation of immune-suppressive Tregs, promote macrophage-mediated defense, and expedite sarcoma cell apoptosis [51]. However, as mentioned above, the polarization of M1 into M2 occurs during tumor progression. Thus, the immunosuppressive M2-like subtype becomes the dominant phenotype in the late stage. M2 TAMs transfer exosomal molecules, including microRNA and lncRNA to promote the survival, proliferation, and migration of sarcoma cells [52, 53]. In osteosarcoma, exosomal lncRNA LIFR-AS1 from M2 TAMs acted as a miR-29a sponge to restrain apoptosis and promote invasion of recipient tumor cells [53].

**Table 2:** Effect of TME-derived exosomes on the sarcoma cells

TME cells	Sarcoma type	Exosomal cargos	Mechanisms	Clinical significance	References
TAMs	OS	\	Promote anti-tumor response; activate caspase-3 and -7	Progression↓	[51]
TAMs	OS	miR-221-3p	Activate JAK2/STAT3 pathway	Progression and metastasis↑	[52]
TAMs	OS	lncRNA LIFR-AS1	Activate miR-29a/NFIA axis	Progression and metastasis↑	[53]
MSCs	OS	\	Decrease hsa-miR-195 and hsa-miR-124, increase hsa-miR-148a	Progression and metastasis↑	[60]
MSCs	OS	miR-21-5p	Activate PI3K/Akt/mTOR pathway	Progression↑	[61]
ADSC	OS	\	Up-regulate COLGALT2, vimentin, and MMP2/9	Progression and metastasis↑	[62]
MSCs	OS	\	Activate Hedgehog pathway	Progression↑	[63]
MSCs	OS	miRNA-208a	Activate ERK1/2 pathway	Progression and metastasis↑	[64]
MSCs	ATRT	miR-155	Down-regulate SMARCA4	Metastasis↑	[65]
MSCs	OS	lncRNA PVT1	Increase ERG level	Progression and metastasis↑	[66]
CAFs	OS	lncRNA SNHG17	Activate miR-2861/MMP2 axis	Progression↑	[71]
OS cells	OS	uPA	Activate uPA/uPAR axis	Metastasis↑	[69]
EWS cells	EWS	miR-210	Down-regulate CASP8AP2	Progression↑	[28]
OS cells	OS	hsa-circ-103801	Reduce cisplatin sensitivity, inhibit apoptosis, and increase MRP-1 and P-glycoprotein expression	Chemoresistance↑	[91]
OS cells	OS	MDR-1 mRNA, P-glycoprotein	Increase MDR-1 mRNA, P-glycoprotein	Chemoresistance↑	[96]

TAMs, tumor-associated macrophages; OS, osteosarcoma; miR, microRNA; JAK2, Janus Kinase 2; STAT-3, signal transducer and activator of transcription 3; lncRNA, long non-coding RNA; LIFR-AS1, Leukemia inhibitory factor receptor antisense RNA 1; NFIA, nuclear factor I A; MSCs, Mesenchymal stem/stromal cells; hsa-miR, homo sapiens microRNA; PI3K, phosphoinositide 3-kinase; Akt, AKT8 virus oncogene cellular homolog; mTOR, mammalian target of rapamycin; PIK3R1, phosphoinositide-3-kinase regulatory subunit 1; ADSC, adipose-derived mesenchymal stem cells; COLGALT2, collagen beta (1-O) galactosyltransferase 2; MMP2/9, matrix metalloproteinases-2 and-9; ERK1/2, extracellular signal-regulated kinase 1/2; ATRT, atypical teratoid/rhabdoid tumor; SMARCA4, SWI/SNF Related, matrix associated, actin dependent regulator of chromatin, subfamily A, member 4; PVT1, plasmacytoma variant translocation 1; ERG, ETS-related gene; CAFs, cancer-associated fibroblasts; SNHG17, Small nucleolar RNA host gene 17; MMP2, matrix metalloproteinase 2; uPA, urokinase-type plasminogen activator; uPAR, uPA receptor; CASP8AP2, caspase 8 associated protein 2; MRP-1, multidrug resistance-associated protein-1; MRP-1, multidrug resistance-1

### Exosomes facilitate the immune cell and sarcoma cell interactions for immunoediting and sarcoma metastasis

Cancer immunoediting is the process in which the immune system can both inhibit and promote cancer progression. Cancer immunoediting includes three phases: elimination (immune activation), equilibrium, and escape phase (immunosuppression). Exosomes can mediate immunoediting and sarcoma development via linking communications between sarcoma cells and immune cells (Table 1). The involvement of macrophages has been highlighted above.

**Fig. 4:**
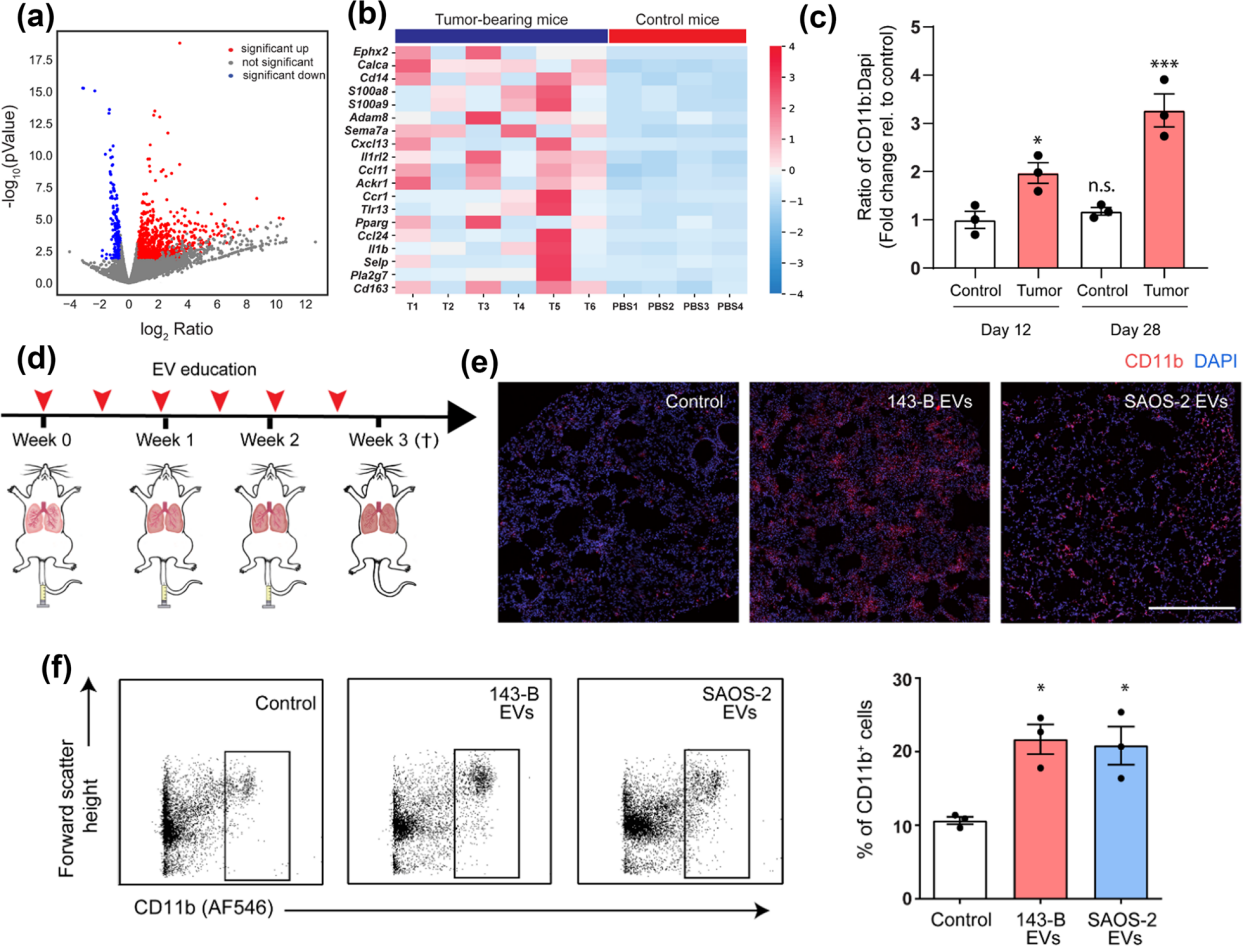
Transcriptomic profiling of pre-metastatic lungs showed an inflammatory response and myeloid cell infiltration, which was promoted by sarcoma-derived extracellular vesicles (EVs). **a** Volcano plot of differentially expressed genes in pre-metastatic lungs. **b** Heatmap representation of up-regulated genes under “inflammatory response” in tumor-bearing mice and controls. Cut-off values of fold change are > 1.5 and FDR < 0.2. **c** Quantification of CD11b+ cells in lungs of controls and tumor-bearing mice (day 12 and day 28). **d** Mice were treated with either PBS, 143-B EVs, or SAOS-2 EVs (10 µg) 2 times a week for 3 weeks. **e** Representative immunofluorescence images of CD11b+ cells (red) and nuclei (blue) in lungs of treated mice. **f** Representative flow cytometric profile (left) and quantification (right) of CD11b+ cells isolated from lungs of treated mice (Reprinted with permission from Ref [56] Copyright © 2020, Cancers, Alekhya Mazumdar et al.)

In the elimination and equilibrium phases, sarcoma-secreted exosomes containing lncRNA FOXP4-AS1 could recruit innate immune cells to TME, including Tregs, activated NK cells, and M1 macrophages [54]. The infiltrating immune cells had either tumor-promoting or tumor-suppressive effects. However, in the escape phase of immunoediting, sarcoma-derived exosomes have been shown to directly impair immune response. These exosomes induced the release of pro-inflammatory cytokines in CD33+ myeloid cells and CD14+ monocytes, and inhibited the differentiation of myeloid cells into dendritic cells [55]. The immature dendritic cells differentiated under this circumstance impeded immune reaction [55]. The immunosuppressive activities included inhibition of T cell proliferation, reduction of IFN-γ release, and induction of IL-10 and IL-6 secretion [55]. Immune cells also participate in exosome-dominated metastatic activities. Osteosarcoma-derived exosomes could recapitulate the infiltration of inflammatory CD11b+ Gr-1+ cells into the pre-metastatic lungs (Fig. 4) [56]. The formation and function of the pre-metastatic niche required a combined involvement of immunocytes and sarcoma-derived exosomes [56].

### Exosomes support the mesenchymal stem cell and sarcoma cell for sarcoma development

Mesenchymal stem cells (MSCs) are pluripotent stem cells with a remarkable capacity for self-renewal and multi-directional differentiation. As non-hematopoietic precursors, MSCs can differentiate into osteoblasts, chondroblasts, adipocytes, etc. [57]. Thus, MSCs contribute to the maintenance and regeneration of connective tissue, especially bone tissue. Exosomes take part in the interactions between sarcoma cells and MSCs.

Sarcoma-originated exosomes educate the recruited MSCs to undergo heterogeneous differentiation into a tumor-promoting phenotype (Table 1). Osteosarcoma-derived exosomes induced LINE-1 hypomethylation, leading to the epigenetic transformation of MSCs into pro-tumorigenic and pro-metastatic cancer-associated fibroblasts (CAFs) phenotype [58, 59]. The transformed MSCs had altered cytokine expression, and considerably over-expression of matrix metalloproteinases, vascular endothelial growth factors, and adhesion molecules [59]. These products contributed to extracellular matrix degrading, angiogenesis, and sarcoma invasion.

**Fig. 5:**
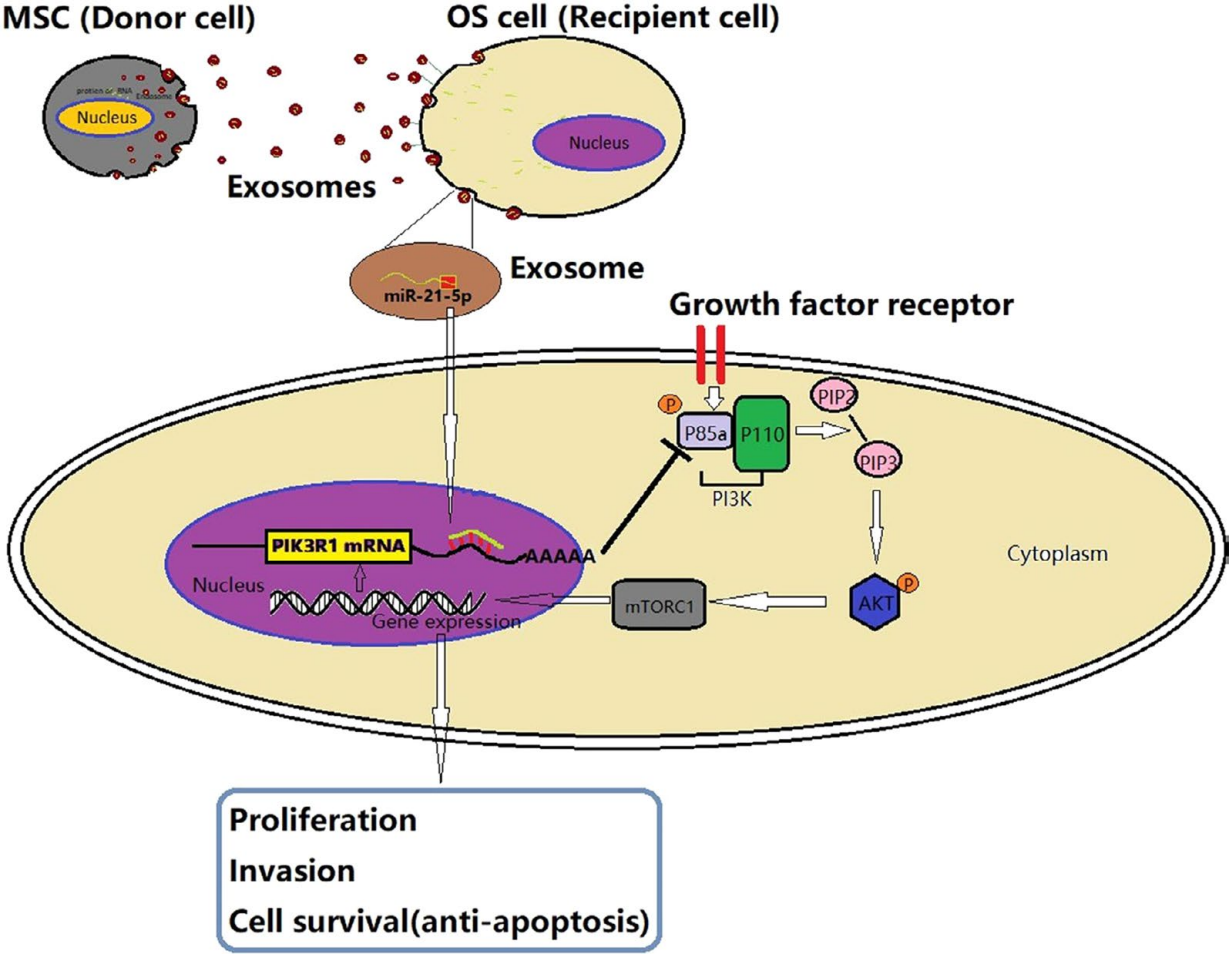
Schematic diagram of the role of exosomal miR-21-5p derived from MSCs in regulating the progression of OS cells. MSCs transported miR-21-5p to sarcoma cells via exosomes. Exosomal miR-21-5p targeted PIK3R1 gene and activated PI3K/Akt/mTOR pathway, thus resulting in sarcoma proliferation, invasion, and survival (Reprinted with permission from Ref [61] Copyright © 2020, Journal of Cellular and Molecular Medicine, Jin Qi et al.)

Correspondingly, MSCs can secrete abundant exosomes and act as paracrine mediators for sarcoma progression in TME (Table 2). MSC-derived exosomes assist the survival and proliferation of sarcoma cells via eluding inhibitory signals from hypoxia and chemotherapeutic agents. Serum-deprived MSCs prevented sarcoma cells from nutrient deprivation-induced death, via exosomes attenuating oxidative phosphorylation and increasing lactate uptake [60]. MSCs also facilitated the resistance of sarcoma to drug-induced apoptosis. Stressed MSCs decreased doxorubicin sensitivity of sarcoma cells through exosomes increasing transporter expression [60]. Meanwhile, MSCs-derived exosomes could also provide stimulatory signals for cancer initiation and development [61–65]. Exosomes carrying oncogenic microRNAs dysregulated proto-oncogenes and tumor suppressor genes in sarcoma cells, leading to the improvement of propagation, invasion, and migration (Fig. 5) [61, 63–65]. For instance, MSCs-derived exosomal miR-208a negatively targeted programmed cell death 4 to activate the ERK1/2 signaling pathway, thereby increasing the viability, clonogenicity, and migration of osteosarcoma cells [64]. The resultant increase in aggressiveness could be partly explained by the exosome-induced epithelial–mesenchymal transition and growth factor secretion [60, 62]. Additionally, MSC-derived exosomes also exerted pro-tumor effects through transporting oncogenic lncRNA PVT1 [66]. The exosomal PVT1 increased the level of transcription factor ERG in osteosarcoma cells [66]. ERG was previously found to be linked with bone matrix formation and sarcoma progression via activating TNSALP transcription [66].

### Exosomes assist the fibroblast and sarcoma cell interactions for sarcoma invasive growth

CAFs are actively involved in cancerous lesions through remodeling matrix, promoting sarcoma progression, and assisting metastatic niche formation. Exosomes mediate the interactions between CAFs and sarcoma cells to support sarcoma expansion.

Sarcoma cells release exosomes to alter fibroblast phenotype, thereby promoting tumor infiltrative and metastatic activities (Table 1). The malignant transformation of neighbouring normal fibroblasts contributed to sarcoma local invasion [67, 68]. The surrounding fibroblasts absorbed exosomes from aggressive sarcoma cells and converted into a tumor-like phenotype [67, 68]. The resulting fibroblasts had enhanced proliferation and survival ability, and acquired capability to grow in an anchorage-independent manner [68]. More importantly, these adjacent transformed fibroblasts facilitated tumor growth into surrounding soft tissues, and led to frequent recurrence of infiltrative myxofibrosarcoma [67]. In addition, the activation of distant fibroblasts is one of the early events in sarcoma metastasis. Sarcoma-derived exosomes drive metastatic phenotype transformation of neighbouring sarcoma cells [69]. Meanwhile, the fibroblasts were recruited to the pre-metastatic lung by sarcoma-derived exosomes [70]. The lung fibroblasts internalized exosomes and differentiated into pro-metastatic myofibroblasts [70]. The acquisition of metastatic property of sarcoma cells and accumulation of lung fibroblasts, together with their conversion into myofibroblasts supported pulmonary metastasis of osteosarcoma [69, 70].

CAFs-derived exosomes also in turn boost the malignant behaviors of sarcoma (Table 2). The exosomes from CAFs carried lncRNA SNHG17, which up-regulated the expression of neoplastic matrix metalloproteinases in sarcoma cells [71]. Thus, the physical barrier was disrupted, and the invasive and metastatic growth of sarcoma was enhanced [71].

### Exosomes expediate the endothelial cell and sarcoma cell interactions for angiogenesis

Angiogenesis refers to the ability of organisms to form neovasculature based on the original vasculature. As growing beyond its vascular supply, sarcoma mass forms hypoxic gradients and even a severely hypoxic core. Hypoxia induces an intricate intracellular signaling network in sarcoma cells which includes HIF, MAPK, PI3K, and NFĸB pathways [28, 72]. These signaling pathways are involved in the regulation of cell proliferation, metabolism, apoptosis, migration, stemness, and inflammatory response [28, 72]. For example, the elevation of miR-210 level in exosomes was dependent on HIF-1α stabilized expression in parental hypoxic cells [28]. Exosomal miR-210 was delivered to normoxic cells to promote sphere formation and enhance stemness via down-regulating proapoptotic CASP8AP2, which is pivotal to the survival of stem-like cells [28]. Although sarcoma cells in the hypoxic niche can directly deliver exosomes to nearby normoxic sarcoma cells for the acclimatization to hypoxia, the formation of neovasculature is a requirement for sarcoma growth [28]. These vessels funnel nutrients and waste metabolites from the sarcoma core, enable immune cell extravasation, and provide opportunities for tumor hematogenous metastasis.

Sarcoma cells encourage the initiation and development of angiogenesis by targeting endothelial cells in an exosome-mediated manner (Table 1). Exosomes increased the levels of angiogenic factors in TME. Sarcoma delivered microRNAs into endothelial cells through exosomes to increase angiogenic factors expression, including VEGF-A, IL-6, and IL-8 [42]. These angiogenic factors could induce neovascularization, regulate endothelial integrity, and modulate vascular permeability [42]. Besides, exosomes from sarcoma cells directly enhanced the reactivity of endothelial cells to these pro-angiogenic molecules. LncRNA and microRNA in sarcoma-derived exosomes up-regulated sensitivity and tube formation capacity of endothelial cells, which were prerequisites for the function of angiogenic factors [42, 73]. The combination of endothelial responsiveness and biosynthesis of pro-angiogenic substances leads to active angiogenesis. The hypoxia-induced dysfunctional vascularization and acquisition of epithelial-to-mesenchymal transition phenotype contribute to the promotion of tumor cell mobility and metastasis.

## Clinical applications of exosomes in sarcoma

**Fig. 6:**
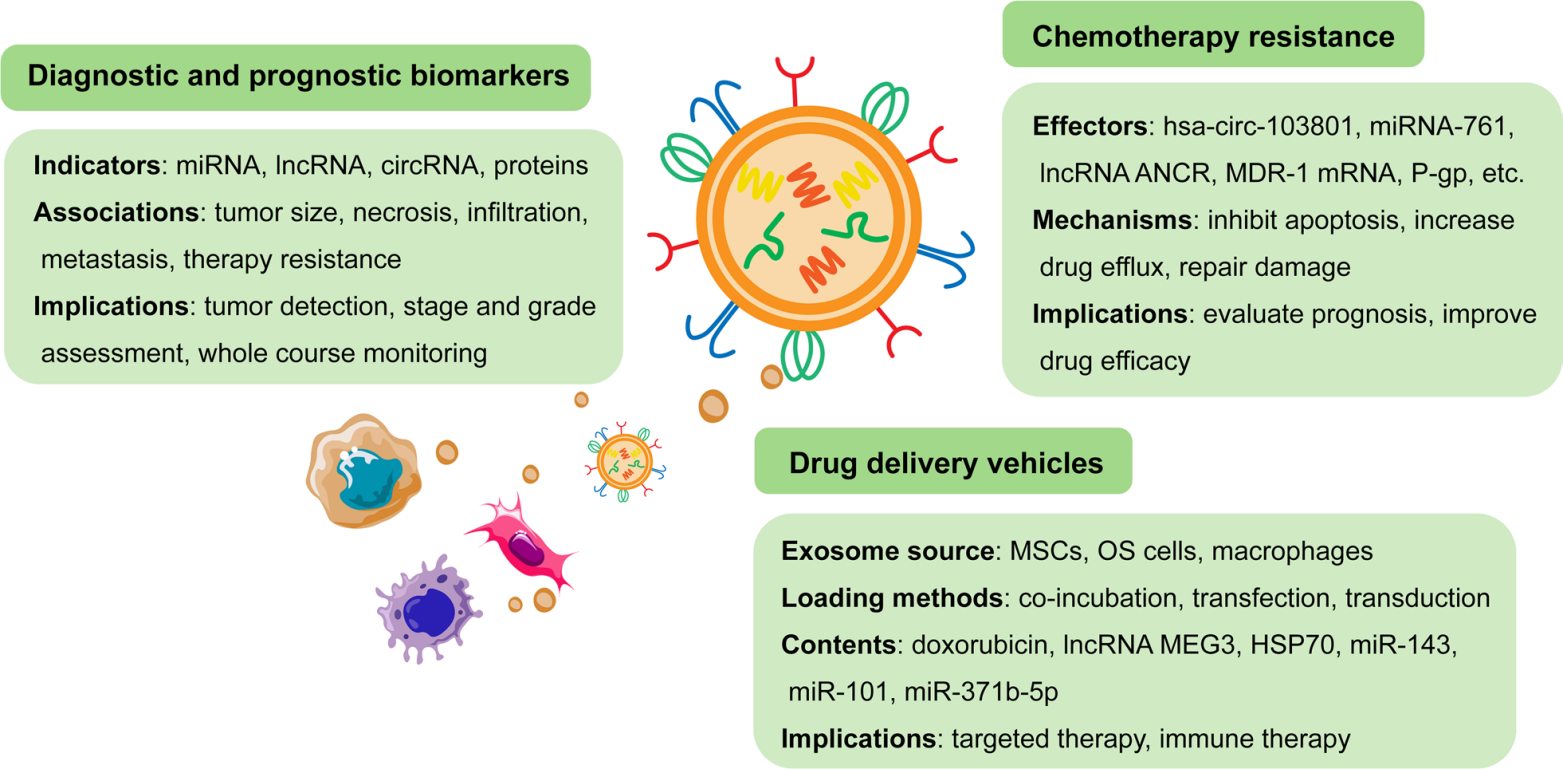
Clinical applications of exosomes in sarcoma management

TME plays an integral part in sarcoma biology, participating in sarcoma initiation, development, and response to treatment. With the in-depth research in this emerging field, the potential clinical value of exosomes in TME has been gradually excavated. The molecular features of sarcoma-derived exosomes mirror many of the molecular features of the sarcoma they originate from, reflecting the physiological status of the sarcoma cells. Meanwhile, exosomes in TME are critical mediators in sarcomagenesis, affecting tumor progression and metastasis. Therefore, exosomes have potential roles in early diagnosis, prognosis prediction, chemosensitivity evaluation, and targeted therapy in sarcoma, which might represent an advancement in precision medicine (Fig. 6).

### Exosomes as diagnostic and prognostic biomarkers

Except for tumor biopsy and radiographic imaging, no clinically relevant indicator is available for the timely detection of sarcoma. As discussed above, sarcoma-derived exosomes are enriched with microRNAs, lncRNAs, circRNAs, and proteins, which are more abundant in sarcoma cells than in normal cells. The similarities between exosomes and the parental cells exhibit the potential of exosomes as biomarkers. The detection of exosomes will assist in tumor burden assessment, therapeutic responsiveness evaluation, and recurrence monitoring.

**Fig. 7:**
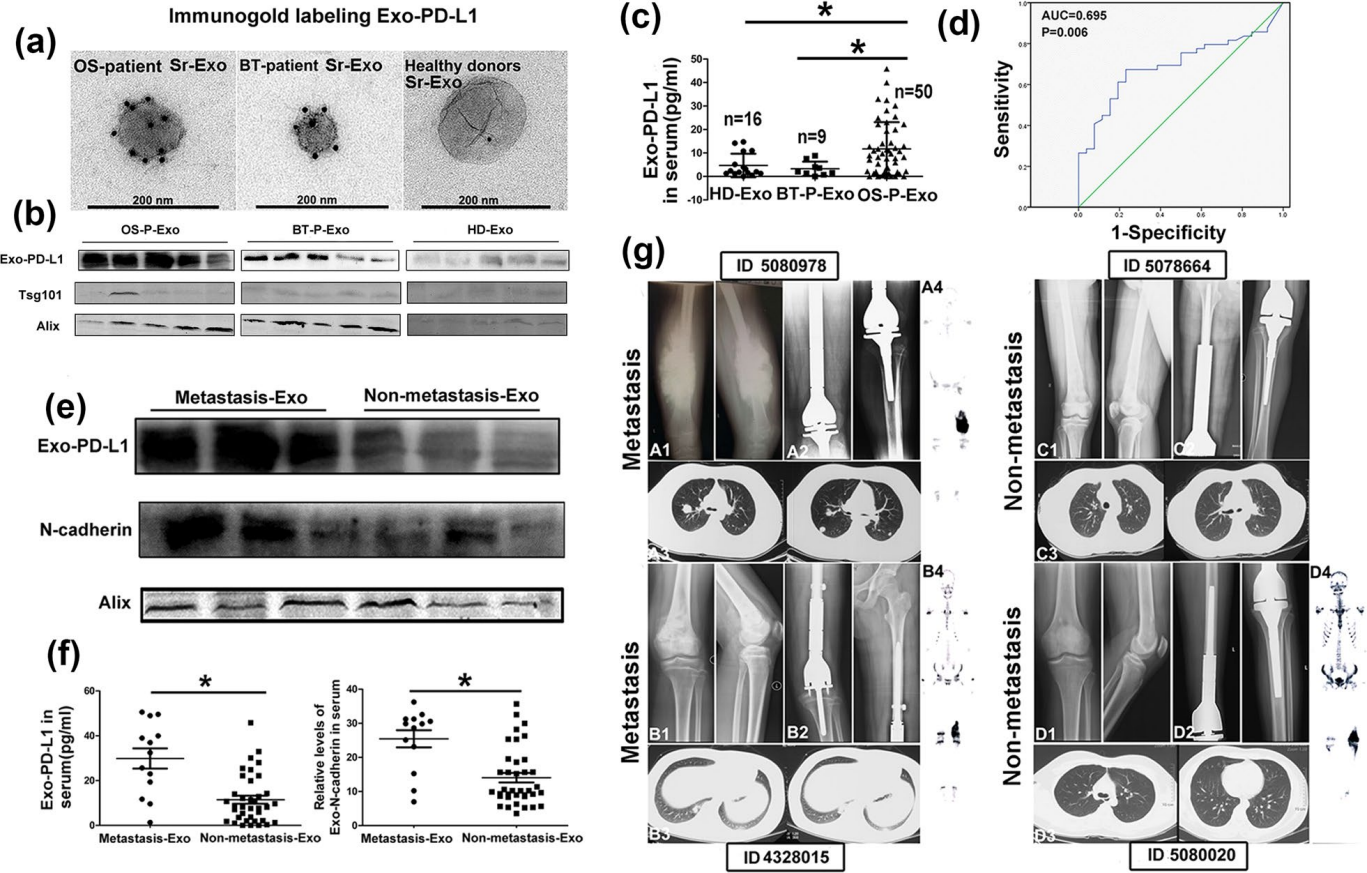
Higher level of exosomal PD-L1 in OS patients, especially those with pulmonary metastasis, compared to healthy donors and patients with benign tumor (BT). **a** A TEM image of exosomes immunogold-labelled with PD-L1 antibody. **b** Western blot analysis of PD-L1 expression in exosomes. **c** The levels of PD-L1 in serum exosomes from healthy donors and patients with OS and BT by ELISA. **d** ROC analysis showed that the AUC of serum-derived exosomal PD-L1 was 0.695, which could distinguish OS patients from healthy controls. **e, f** Higher expression of exosomal PD-L1 and N-cadherin in OS patients with metastasis than those without metastasis via western blot and ELISA analysis. **g** Radiological images of 4 OS patients with and without pulmonary metastasis (Reprinted with permission from Ref [86] Copyright © 2020, Journal of Nanobiotechnology, Jun Wang et al.)

Each sarcoma is characterized by its specific nucleic acid or protein profile, which is the basis of the molecular diagnosis of sarcoma. Liquid biopsy profiling of plasma exosomes in sarcoma has been found to show great potential for tumor detection, and stage and grade assessment (Table 3). High serum levels of exosomes containing specific microRNAs are related to sarcoma status [67, 74–81]. High-throughput sequencing identified that 57 micorRNAs in serum exosomes were differentially expressed in osteosarcoma patients and controls with 20 expression being up-regulated and 37 being down-regulated [77]. The increased exosomal miR-195-3p could promote the proliferation and invasion of sarcoma cells, which further supported its significance in sarcoma detection [77]. Serum levels of exosomal miR-1260b were high in ten patients with infiltrative myxofibrosarcoma, which were more dominant in preoperative patients’ samples than those in postoperative [67]. The circulating exosomal miR-1260b levels were associated with tumor burden and the infiltrative ability of myxofibrosarcoma [67]. Serum-based circulating microRNAs are expected to serve as non-invasive and repeatable liquid biopsy indicators for tumor monitoring in sarcoma patients. Proteomic investigations of circulating exosomes revealed that some proteins were helpful in indicating tumor progression and metastasis [82–85]. For example, the level of serum exosomal PD-L1 and N-cadherin was higher in osteosarcoma patients, especially in the ones with pulmonary metastasis, than that in healthy controls [86]. The determination of cut-off values of these proteins proved to be useful in the identification and clinical staging of osteosarcoma patients (Fig. 7) [86]. Currently, based on mass spectrometry and proteome profiles, thousands of plasma exosomal proteins can be captured from a trace sample for the evaluation of osteosarcoma lung metastasis [87, 88]. Seven exosomal proteins were selected to differentiate patients with osteosarcoma from healthy controls and further distinguish between those with lung metastasis and non-lung metastasis [87]. The exosome-derived fusion transcripts and lncRNAs are also candidate diagnostic biomarkers for sarcoma [89, 90]. A deep analysis of these exosomal components and their levels will support an accurate diagnosis of sarcoma.

**Table 3:** Studies on the diagnostic and prognostic values of exosomes

Sarcoma type	Exosome source	Exosomal cargos	Sample source	Clinical application	References
MFS	Serum	miR-1260b	15 patients, 5 non-sarcoma patients, 9 healthy controls	Diagnostic biomarker	[67]
DSRCT	Serum	miR-34a-5p, miR-22-3p, miR-324-5p	3 patients, 4 healthy controls	Diagnostic biomarker	[74]
OS	Serum	miR-195-3p	25 patients, 10 healthy controls	Diagnostic biomarker	[77]
OS	Serum	miRNAs	5 patients	Diagnostic biomarker	[78]
DDLPS	Serum, tissue	miR-1246, -4532, -4454, -619-5p, and -6126	22 OS, 17 DDLPS, 3 EWS patients	Diagnostic biomarker	[79]
SS	Serum	miR-92b-3p	12 SS patients, 12 benign tumor patients, 12 healthy controls	Diagnostic biomarker	[80]
RTK	Serum	miR-214-3p	10 patients, 10 healthy controls	Diagnostic biomarker	[81]
OS	Serum	Proteins	8 patients, 5 controls with fracture, 5 healthy controls	Diagnostic biomarker	[83]
EWS	Serum	CD99/MIC2, NGFR	10 patients, 6 healthy controls	Diagnostic biomarker	[84]
GIST	Serum	KIT, SPRY4	9 patients, 9 healthy controls	Diagnostic and prognostic biomarker	[85]
OS	Serum	PD-L1, N-cadherin	70 OS patients, 9 benign tumor patients, 22 healthy controls	Diagnostic biomarker	[86]
OS	Serum	Proteins	20 lung metastasis patients, 20 non-metastasis patients, 12 healthy controls	Diagnostic biomarker	[87]
ARMS, SS	Serum	Fusion transcripts	65 ARMS patients, 15 SS patients	Diagnostic biomarker	[89]
OS	Serum, tissue	lncRNA CASC15	Sera from 5 patients and healthy controls; 30 OS specimens, 30 normal bone tissues	Diagnostic biomarker	[90]
OS	Serum	hsa-circ-103801	43 patients, 5 healthy controls	Prognostic biomarker, chemoresistance↑	[91]
OS	Tissue	lncRNA SNHG17	5 pairs of tumor and tumor-free tissues	Prognostic biomarker	[75]
OS	Serum, tissue	miR-25-3p	Sera from 10 patients and 10 healthy controls; 45 biopsy specimens	Prognostic and diagnostic biomarker	[76, 93]
OS	Serum, tissue	SENP1	Sera from 146 patients; 60 pairs of tumor and tumor-free tissues	Prognostic biomarker	[92]
OS	Serum	Alpha-2-macroglobulin, protein S, complement C2	\	Prognostic biomarker, chemoresistance↑	[94]
OS	Serum	lncRNA ANCR	10 patients, 10 chemosensitive controls	Prognostic biomarker, chemoresistance↑	[95]

MFS, myxofibrosarcoma; miR, microPNA; DSRCT, Desmoplastic small round cell tumor; OS, osteosarcoma; DDLPS, dedifferentiated liposarcoma; EWS, Ewing’s sarcoma; SS, synovial sarcoma; RTK, rhabdoid tumor of the kidney; NGFR, nerve growth factor receptor; GIST, gastrointestinal stromal tumor; KIT, tyrosine kinase receptor; SPRY4, Sprouty homolog 4; PD-L1, programmed death-ligand 1; ARMS, alveolar rhabdomyosarcoma; lncRNA, long noncoding RNAs; CASC15, cancer susceptibility 15; hsa, homo sapiens; SNHG17, Small nucleolar RNA host gene 17; SENP1, sentrin SUMO-specific protease 1; ANCR, Angelman syndrome chromosome region

Accumulating studies have discovered that the molecular types and expression levels inside exosomes vary with disease progression, making them well-suited to assess prognosis and therapeutic efficacy. The levels of exosomal cargos, including lncRNAs, circRNAs, microRNAs, and proteins, had clinicopathological relevance in tumor size, necrosis rate, pulmonary metastasis, and therapy resistance (Table 3) [71, 76, 86, 91–95]. Sarcoma patients with higher expression of these exosomal contents in tissues and sera showed higher metastasis and recurrence rates, and shorter survival time [71, 86, 92, 93]. SNHG17 transcript level in the osteosarcoma tumor tissues was approximately 3 times higher than that in the normal tissues, and SNHG17 was mainly encapsulated in exosomes [71]. Patients with high expression of SNHG17 were significantly associated with poor prognosis [71]. The detection of circulating exosomes may be more valuable than that of tissue exosomes in clinical applications. A proteomic signature to discriminate between poor and good prognosis with high accuracy was revealed via using mass spectrometry to compare serum exosomes with different disease-free interval [94]. The expression levels of exosome-derived sentrin SUMO-specific protease 1 (SENP1) in patients’ plasma were significantly related to tumor stage, surgical stage, and overall survival of patients [92]. Patients with higher expression of plasma exosome-derived SENP1 had worse disease-free survival and overall survival [92]. The area under the receiver operating characteristic curve of plasma exosomal SENP1, as 1- and 3-year disease-free survival biomarkers, was 0.90 and 0.96, respectively [92]. The plasma exosome-derived SENP1 was superior to plasma SENP1 as a prognostic biomarker [92]. Up-regulated level of specific molecules in the serum exosomes from patients could also imply that sarcoma cells were resistant to chemotherapy and those patients might have a shorter survival time [91].

### Exosomes as mediators of chemotherapy resistance

Drug resistance development is one of the crucial reasons for chemotherapy failure and poor prognosis in sarcoma. Exosomes typically function in the export of waste metabolites and signaling molecules from parental cells. Drug-resistant sarcoma cells are capable of shuttling chemotherapeutic agents out of cells, and delivery drug resistance molecules to tumor cells in TME via exosomes. Therefore, exosomes hold important roles in chemotherapy resistance of sarcoma.

**Fig. 8:**
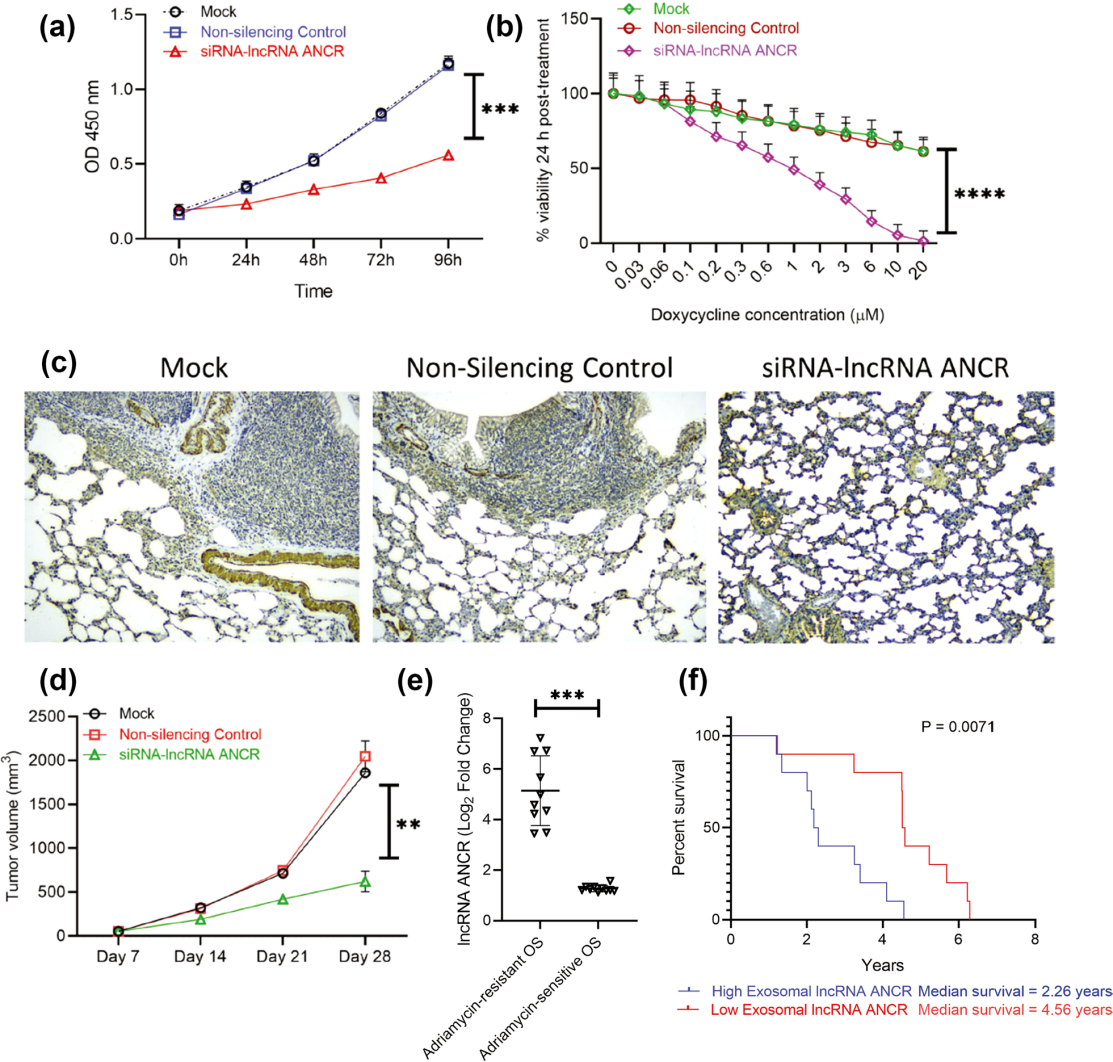
Exosomal lncRNA ANCR dictates in vitro sensitivity of sarcoma to doxycycline, expression of which is critical for drug resistance, tumor progression, and overall survival of patients. **A** Relative growth rate of the drug resistant cells mock-transfected, or transfected with non-silencing control siRNA, or siRNA targeting DANCR were quantified using CCK8 assay. **b** Cell viability of the differently transfected drug resistant cells after treated with doxycycline. **c** Representative IHC staining of osteocalcin. Nude mice were injected with sarcoma cells pre-treated with exosomes and added to sarcoma cells. All mice were treated with doxycycline orally once every 3 days for 4 weeks. Knockdown of DANCR made sarcoma cells sensitive to doxycycline and inhibited lung metastasis. **d** The tumor growth rate. **e** Expression of DANCR in OS patients sensitive or resistant to Adriamycin (n = 10 each) via using RT-qPCR. **f** Kaplan–Meyer survival curve showed significantly higher overall survival in patients with lower ANCR expression (median survival 4.56 years) than those with higher ANCR expression (median survival 2.26 years; P = 0.0071) (Reprinted with permission from Ref [95] Copyright © 2022, Frontiers in oncology, Xin Hu et al.)

Exosomes have been shown to transfer circRNAs, microRNAs, lncRNAs, mRNAs, and proteins from drug-resistant cells to drug-sensitive cells, thereby inducing primary drug resistance and multiple drug resistance [91, 94–97]. For instance, the multidrug-resistant osteosarcoma cells released exosomes containing multidrug resistance-1 mRNA and P-glycoprotein [96]. The exosomes could be taken up into secondary cells, facilitating the convey of doxorubicin-resistant capacity [96]. Exosomes highly enriched with hsa_circ_103801 derived from cisplatin-resistant cells also reduced drug sensitivity via inhibiting apoptosis and increasing the expression of multidrug resistance-associated protein 1 and P-glycoprotein [91]. Extracellular miR-761 from drug-resistant cells targeted three proteins, including thyroid hormone receptor interactor 6, lamin A/C, and NAD-dependent protein deacetylase sirtuin-3 [97]. Knockdown of any of these proteins in recipient cells could confer increased resistance to chemotherapeutic agents [97]. Briefly, the establishment of chemoresistance in sarcoma cells involves multiple mechanisms, including down-regulating apoptosis, increasing drug efflux, and modulating the expression of multidrug resistance substances [91, 96, 97]. Expression of the exosomal lncRNA ANCR in plasma was associated with the resistance to doxorubicin, and the lncRNA ANCR level was negatively correlated with survival time in osteosarcoma patients [95]. Therefore, detection of exosomes in serum during disease management contributed to the monitor of sarcoma chemosensitivity for better guidance of personalized chemotherapy treatment (Fig. 8) [91, 95].

### Exosomes as potential delivery vehicles of therapeutic agents

The critical functions of exosomes in sarcoma development demonstrate their potential as therapeutic vectors. Exosomes, with phospholipid bilayers and specific ligands, communicate with targeted cells in TME and directly fuse with their cytomembrane, thus increasing the internalization of the encapsulated drugs. An exosome-based delivery system is expected to improve efficacy, reduce side effects, and prolong the half-life of cancer drugs (Table 4).

**Table 4:** Studies on exosomes as potential delivery vectors in sarcoma treatment

Sarcoma type	Target cells	Exosome source	Exosomal cargos	Loading methods	Mechanisms	Loading efficiency	References
OS	Sarcoma cells	MSCs	Doxorubicin	Co-incubation	Nucleic acid synthesis ↓	12%	[98]
OS	Sarcoma cells	MSCs	miR-143	Lipofection	Cell migration ↓	Not mentioned	[99]
OS	Sarcoma cells	OS cells	lncRNA MEG3	Lipofection	Sponging miR-185-5p	Not mentioned	[100]
FS	Immune cells	Macrophages	HSP70	Incubation at 42℃	Immune response ↑	Not mentioned	[101]
OS	Sarcoma cells	MSCs	miR-101	Lentiviral transduction	Cell migration ↓	About 48%	[108]
OS	Sarcoma cells	OS cells	miR-371b-5p	Co-incubation	Cell proliferation and migration ↓	Not mentioned	[109]

OS, osteosarcoma; MSCs, mesenchymal stem cells; miR, microRNA; lncRNA, long non-coding RNA; FS, fibrosarcoma; HSP70, heat shock protein 70

**Fig. 9:**
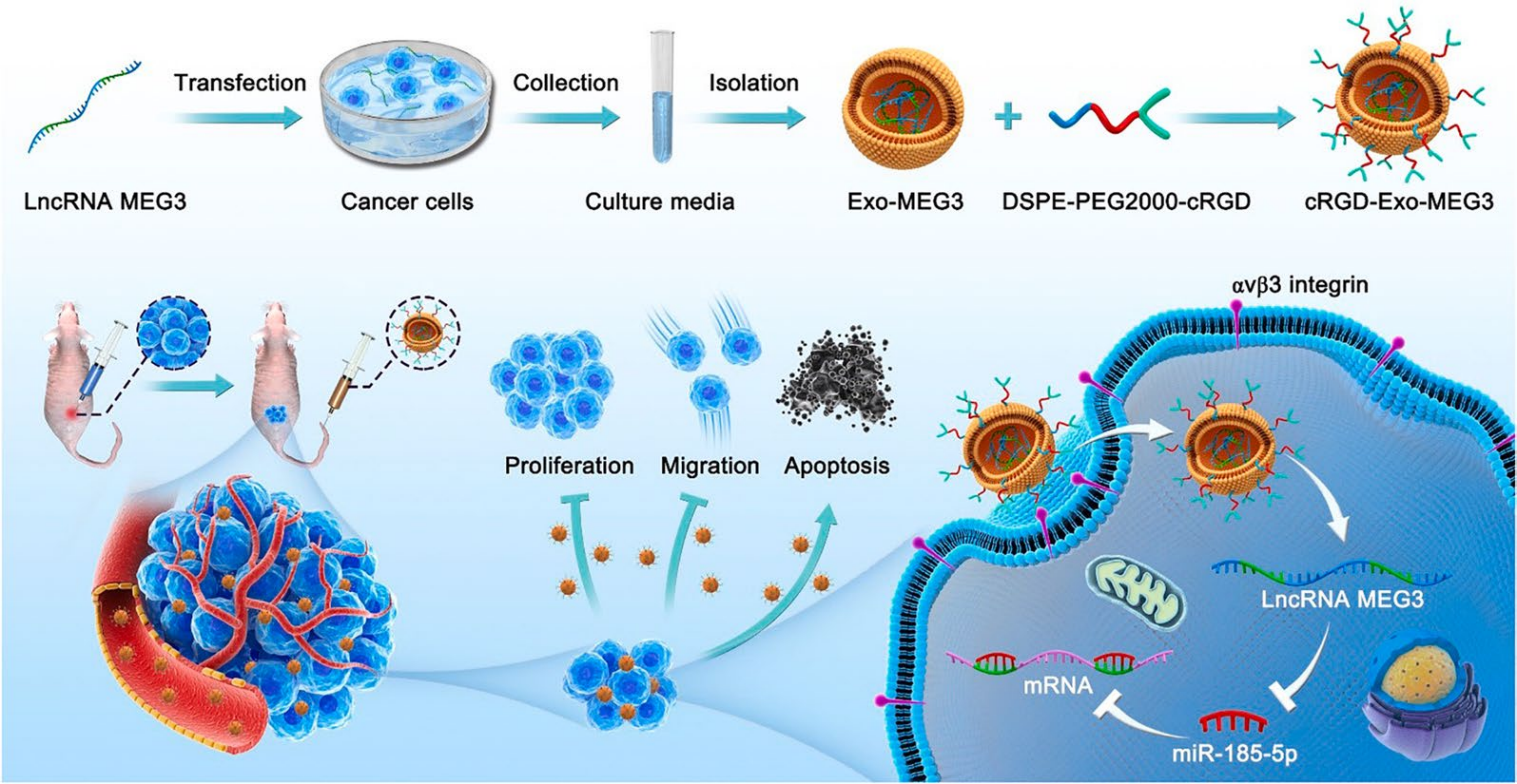
Schematic diagram of the synthesis of cRGD-Exo-MEG3 and its application as a targeted lncRNA MEG3 delivery vectors for osteosarcoma treatment (Reprinted with permission from Ref. [100] Copyright © 2022, Journal of Controlled Release, Xin Huang et al.)

Bioactive molecules can be loaded into exosomes by directly modifying isolated exosomes or indirectly manipulating producer cells. The exosomes extracted from MSCs were loaded with doxorubicin by co-incubation with doxorubicin-HCl [98]. Doxorubicin enveloped in the exosomes showed enhanced cellular uptake efficiency and tumor-killing efficacy with lower cytotoxicity to normal cells than free drug [98]. The producer MSCs could also be engineered via transfection of miR-143 intracellularly to release miR-143-loaded exosomes [99]. The miR-143-loaded exosomes significantly reduced the migratory potential of sarcoma cells [99]. Similarly, the modified tumor cells produced lncRNA MEG3-loaded exosomes [100]. The isolated exosomes were further co-incubated with micelles containing cRGDyK to prepare cRGD-conjugated exosomes for enhanced tumor-targeting [100]. The cRGD peptide can specifically bind to α_v_ β_3_ integrin, which is up-regulated on cancer cells and activated endothelial cells of growing vessels (Fig. 9). In addition to acting as drug vehicles, exosomes can also assist immunization therapy. The TAMs-derived exosomes enriched in heat shock protein 70 were a viable immunoadjuvant in sarcoma immunotherapy [101]. The number of sarcoma cells notably decreased after vaccinating HSP70-loaded exosomes [101]. Exosomal membrane-bound Staphylococcal enterotoxin B had a synergistic effect with HSP70, leading to the activation of T cells and secretion of cytokines [101].

To apply exosomes as delivery vectors, it is necessary to understand the distribution mode and location of exogenously administered exosomes in vivo. The administered exosomes mainly accumulated in the liver, spleen, lung, gastrointestinal tract, and bone marrow [102]. The surface of exosomes can be modified with specific molecules that can selectively bind to sarcoma cells for better accumulation at the tumor site. B16-BL6 exosomes were promptly cleared from the circulation after systemic administration with a circulating half-life of 2 min, and little could be detected in the serum at 4 h after injection [103]. From the pharmacokinetic profile of exosomes, intravenously administered exosomes had a half-life of 2–30 min [104]. Macrophage-mediated phagocytosis was responsible for the rapid clearance, which strongly reduced the number of available particles and limited exosome accumulation in the lesions. Engineering exosomes with antiphagocytic molecules could evade immune recognition and thus increase the circulating half-life and bioavailability of exosomes. The candidate molecules comprised CD47, CD24, CD31, β2M, and PD-L1 [105]. For instance, CD47 interacts with the immune inhibitory receptor SIRPα and can neutralize detection by phagocytic cells. The CD47-SIRPα axis inhibited macrophage activation and activated the ‘don’t eat me’ signal [105]. The exosomes with high expression of CD47 could be highly detected for a longer time. CD47 was generally up-regulated in exosomes derived from MSCs, making them suitable production cells [105].

## Conclusion and future direction

Significant evidence supports that TME plays critical roles in the initiation and progression of sarcoma. Exosomes are key components of the TME and act as messengers of intercellular communications. This review brings together information on the roles of exosomes in the bidirectional crosstalk between sarcoma cells and TME cells, as well as the clinical value of exosomes. Although existing studies cannot fully elaborate the functions of exosomes, these studies highlight the increasing significance and potential applications of exosomes in sarcoma microenvironment.

However, some critical problems remain unsolved. The term, exosomes, has been widely applied to various kinds of extracellular vesicles, muddying the field and causing the research to be sometimes treated with skepticism. Exosomes can be defined as a subtype of extracellular vesicles, which are released from cells upon fusion of the multivesicular body with the plasma membrane. But this definition is not helpful for the purity and uniformity assurance of exosomes. The heterogeneity of exosomes impedes the identification of exosomes and induces intricate biological reactions, which hinders a comprehensive understanding of their biogenesis, contents, biodistribution, and roles in the TME. The size and amount of biophysically similar extracellular vesicles also make exosomes difficult to be obtained as relatively pure preparations and to characterize properly [106]. Ascribing the exquisite and specific functions of exosomes requires concrete information reporting beyond the mere description of activities in a potentially contaminated and heterogeneous preparation [106]. Many techniques have been developed to extract exosomes, such as ultracentrifugation, tangential flow filtration, ultrafiltration devices, and size exclusion chromatography. Some isolation methods may degrade the structural integrity and functionality of exosomes. There is still no established exosome isolation method as a gold standard. These factors are the obstacles in the exploration of exosome roles in tumor development and the search for specific clinical indicators. Fundamental research should be conducted to reveal the unique characteristics of exosomes to distinguish them from other extracellular vesicles and identify specific exosome populations.

In terms of exosome therapeutic applications in sarcoma, the primary consideration is the cell source that will be used as producer cells, including dendritic cells, MSCs, and patient-derived tumor cells. MSCs may be a major candidate, because MSC-derived exosomes with high CD47 expression have a long half-life. Therapeutic exosomes might also be extracted from different plants, such as ginger, grapes, and lemons [107]. However, the immunotoxicity of exosomes needs to be taken as important. Exosomes extracted from allogenic or heterologous cell sources may elicit immune responses. Although patients injected with trillions of exosomes via blood transfusion do not show immune-related toxicity, the immunogenicity of exosomes developed for sarcoma therapy needs to be thoroughly evaluated. A third limitation in exosome-based treatments is the heavy workload while unsatisfactory yield during exosome production, as well as low loading and delivery efficiency. The clinical breakthrough may depend on the advances in exosome-mimetics and genetic engineering.

These understandings of exosomes in TME of sarcoma may be enhanced through the following aspects: (1) intensive exploration of biological properties of exosomes; (2) searching for more effective methods in exosome detection and analysis; (3) comprehension of the dynamic cellular interplay in TME; (4) discovery of available biomarkers and vehicles with targetability; (5) exploitation of valid approaches to load exosomes with markers and agents; (6) optimization of the target delivery mechanisms of exosomes; (7) conducting clinical trials to confirm existing hypotheses.

## Data Availability

Not applicable.
